# Quantification of Metamorphopsia Using a Smartphone-Based Hyperacuity Test in Patients With Idiopathic Epiretinal Membranes: Prospective Observational Study

**DOI:** 10.2196/60959

**Published:** 2025-04-17

**Authors:** Daria Amon, Christoph Leisser, Andreas Schlatter, Manuel Ruiss, Caroline Pilwachs, Natascha Bayer, Josef Huemer, Oliver Findl

**Affiliations:** 1 Vienna Institute for Research in Ocular Surgery, A Karl Landsteiner Institute, Hanusch Hospital, Vienna, Austria; 2 NIHR Biomedical Research Center at Moorfields Eye Hospital NHS Foundation Trust and UCL Institute of Ophthalmology, 11-43 Bath Street Lower Ground Floor, London, EC1V, United Kingdom, 44 207 253 3411; 3 Department of Ophthalmology and Optometry, Kepler University Hospital, Linz, Austria

**Keywords:** mobile health, smartphone, telemedicine, Alleye, M-chart, metamorphopsia, epiretinal membrane, vitrectomy with membrane peeling, visual acuity, home monitoring, hyperacuity test, hyperacuity, surgical intervention, distorted vision, vision, ocular pathology, ocular, retinal, retina, surgery, macular degeneration, tomography, vitrectomy, ophthalmology

## Abstract

**Background:**

Quality of vision in patients with idiopathic epiretinal membranes (iERMs) is closely linked to distorted vision (metamorphopsia), which is often underestimated in clinical settings. Current surgical decision-making relies heavily on visual acuity and optical coherence tomography findings, which do not adequately reflect the patient’s functional vision or the severity of metamorphopsia. There is a clinical need for tools that can reliably quantify this symptom to improve patient outcomes and streamline care pathways.

**Objectives:**

This study is the first to assess the use of a novel smartphone-based hyperacuity test (SHT) in quantifying metamorphopsia before and after surgical intervention for iERMs, comparing it with a conventional printed chart.

**Methods:**

This prospective observational study included 27 patients with iERMs with symptomatic metamorphopsia detected on the Amsler grid scheduled for vitrectomy with membrane peeling. The SHT (Alleye, Oculocare Medical Inc) and the horizontal (MH) and vertical (MV) M-chart (Inami & Co, Ltd) tests were performed 3 times before and 3 months after surgery. Pre- and postoperative metamorphopsia scores, changes in distance-corrected visual acuity, optical coherence tomography biomarkers, and subjective perception of metamorphopsia were evaluated.

**Results:**

The mean SHT score significantly (*r*=0.686; *P*<.001) improved from 55.2 (SD 18.9) before surgery to 63.5 (SD 16.3) after surgery while the improvement of the M-chart scores were insignificant (MH *r*=0.37, *P*=.06; MV *r*=0.18, *P*=.36). Pre- and postoperative SHT scores showed very weak and insignificant correlations with the MH, MV, and MH+MV scores. Both metamorphopsia tests showed good reliability (intraclass correlation coefficients >0.75).

**Conclusions:**

The SHT showed a significant improvement in postoperative metamorphopsia scores, indicating that it could be a valuable tool for quantifying visual distortion in patients with iERMs. While discrepancies with M-chart results were observed, both tests demonstrated good reliability. Clinically, the SHT may offer a practical solution for monitoring metamorphopsia and guiding complex surgical decision-making, particularly in telemedicine settings. Its accessibility could improve patient management, potentially enhancing preoperative triaging and reducing unnecessary visits.

## Introduction

An epiretinal membrane (ERM) is a common disorder leading to a decrease in visual acuity and distorted vision, called “metamorphopsia” in later stages [1,2]. ERMs in the majority of cases are idiopathic with no identifiable cause; however, they may be secondary due to an already existing ocular pathology. Metamorphopsia, one of the leading symptoms of ERM, can be detected with the Amsler grid or the metamorphopsia chart (M-chart; Inami & Co, Ltd); however, these tools, including the M-chart, are often not routinely used in clinical practice despite metamorphopsia’s importance in surgical decision-making. Clinicians often do not routinely assess metamorphopsia despite its impact on surgical decisions, relying instead on visual acuity, which often fails to reflect the subjective and functionally significant experience of metamorphopsia. Visualization of the membrane and evaluation of microstructural retinal alterations are best achieved with optical coherence tomography (OCT) but do not fully capture the functional visual disturbances experienced by patients [3]. ERMs are surgically treated via vitrectomy and membrane peeling. The degree of postoperative metamorphopsia is difficult to predict and the quantification of metamorphopsia may be helpful to identify patients who may benefit the most from surgery.

Recently, the emergence of digital health solutions, particularly smartphone-based apps, has introduced new possibilities for accessible and practical tools to assess metamorphopsia. The Alleye app (Oculocare Medical Inc), a smartphone-based hyperacuity test (SHT) with Food and Drug Administration 510 (k) clearance, is the first mobile app to provide a quantitative metamorphopsia result. It is already in use for monitoring the progression of metamorphopsia in retinal diseases such as age-related macular degeneration [4,5]. Compared with the conventional printed M-chart for metamorphopsia quantification, the SHT provides a unified score that supports longitudinal monitoring, examines more retinal axes for a comprehensive assessment, and incorporates gamification to improve patient adherence [4,5]. This may make it a practical tool for continuous care, especially as patients with ERM require long-term monitoring with frequent checkups. The SHT’s remote testing capability may reduce the need for in-person visits and support telemedicine pathways, aligning with modern trends in patient-centered health care. Its relevance in the presence of ERM has yet to be evaluated.

This study represents the first attempt to evaluate the SHT in patients with idiopathic epiretinal membranes (iERM) by correlating the SHT (Alleye app) to the conventional printed chart (M-chart) for metamorphopsia quantification before and after iERM surgery. It aims to explore whether the SHT can serve as a reliable, efficient tool for metamorphopsia measurement in a clinical setting. In addition, the study also investigated the correlation between the metamorphopsia scores and retinal OCT biomarkers as well as subjective perception of metamorphopsia.

## Methods

### Ethical Considerations

The study was approved by the ethics committee of the City of Vienna (approval no. EK21-027-0321) and the Austrian Agency for Health and Food Safety. All research activities were conducted in accordance with institutional and national guidelines and complied with the Declaration of Helsinki and the Good Clinical Practice guidelines of the European Union. This study was registered at ClinicalTrials.gov with the identification number NCT05138315. Written informed consent was obtained from all participants before their enrollment in the study. All participants were fully informed about the nature of the study, and they had the opportunity to opt out at any time. Participant data were pseudonymized and deidentified to ensure confidentiality. Strict protective measures were in place to safeguard all participant information throughout the study. No financial compensation was provided to participants for their involvement in the study and participation was entirely voluntary. No images of identifiable individuals are included in the manuscript.

### Study Design and Patients

This prospective observational study was conducted at the department of ophthalmology of the Hanusch Hospital, Vienna, Austria. The study included 30 eyes of 30 patients who met the inclusion criteria and were scheduled for membrane peeling with vitrectomy for iERM. Eligibility criteria included the presence of iERM, age above 18 years, written informed consent, best distance-corrected visual acuity (DCVA) ≤1.0 logMAR and metamorphopsia detected on the Amsler grid. Patients diagnosed with other macular disorders including age-related macular degeneration or participants who had undergone previous intraocular surgery except for uncomplicated cataract surgery were excluded from the study.

Preoperative examinations and the 3 months postoperative follow-up were performed by the team of the Vienna Institute for Research in Ocular Surgery, including an ophthalmologist (CL). Study-related ophthalmic examinations included slit lamp biomicroscopy, retinal examination, DCVA Early Treatment Diabetic Retinopathy Study (ETDRS), metamorphopsia testing (Amsler grid, SHT, and M-charts), and spectral-domain (SD)-OCT with the CE-certified Spectralis OCT (Heidelberg Engineering). The SD-OCT images were obtained using the fast scan protocol (25 sections, 240 µm, 30° angle, 0.75 D focus, AUTO 71 sensitivity, 100% IR power, OCT Volume mode, and high-speed rate of 8.8/s). Apart from these examinations, slit lamp biomicroscopy and SD-OCT were routinely performed in the outpatient department 1 week and 1 month after surgery.

### Surgical Procedure

All patients included into this study were operated by the same surgeon. Surgery included a 23-gauge pars plana vitrectomy and membrane peeling in all cases. For visualization of the ERM and internal limiting membrane (ILM), chromovitrectomy was performed with a trypan blue and brilliant blue G-based dye (MembraneBlue Dual; DORC). The ERM peeling was performed using an end-gripping forceps. Peeling of the ILM was performed as a second step in all cases where it had not been removed en bloc with the ERM. A restaining has been performed in all patients to identify and peel ILM residues. If air tamponade was needed, a complete fluid-to-air exchange was performed at the end of surgery and patients were advised to postoperative prone positioning for 24 hours. Gas tamponade (SF6 or C3F8) was used only in cases with coexistent peripheral retinal breaks.

### Metamorphopsia Tests

The main outcome variables in this study are the preoperative and 3 months postoperative mean SHT scores and mean M-chart scores. Both the M-chart and the SHT were consecutively performed 3 times before surgery and 3 months after surgery. The mean scores were used for statistical analysis and reliability of the 3 test repetitions was assessed. Detection of metamorphopsia on the Amsler grid was also included. The Amsler test was performed only once before and after surgery, as it provides a simple yes or no answer and was used as an inclusion criterion for the study. During the metamorphopsia examinations the eye not being tested was covered. In case patients needed reading glasses for near vision, they were asked to wear their own glasses or received a suitable near addition. All metamorphopsia examinations were tested with about 30-cm distance from the face and iPad rotation was standardized to a horizontal alignment.

For the Amsler examination, the participants were asked to fixate on the central dot of the grid and evaluate whether the lines are straight and parallel and whether the squares are regular and equal in size. If any of these characteristics were mentioned, the Amsler test was positive.

The M-chart was performed 3 times both in horizontal (MH score) and vertical (MV score) planes. The examiner alternated between the 2 positions to get more objective scores. The test has 19 dotted lines with dot intervals between 0.2º and 2.0º visual angles. The patients were shown these dotted lines beginning with 0º until the minimum visual angle needed to cause the metamorphopsia to disappear being the score.

**Figure 1.**
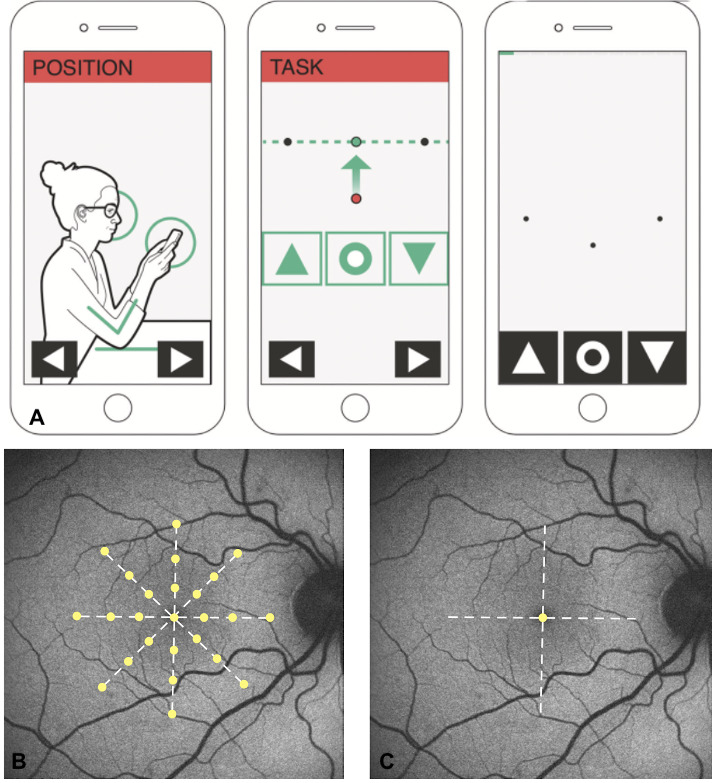
Correct positioning of the smartphone-based hyperacuity test (SHT) and alignment of the central dot on an imaginary straight line between the paracentral points (A) [6]. Four axes examined 3 times by the SHT with the target dot placed at different positions (B) compared with the M-chart with the horizontal and vertical axis and a central fixation dot (C) [7].

The SHT (Alleye app) software can be downloaded on Apple iOS devices or Google Play. The SHT was consecutively performed 3 times and the testing procedure before and after surgery was the same. The patients received an oral explanation of the examination aided by the “training” option on the app. The eye not being tested was covered. The patient held the iOS device (iPad) and the task of the SHT was to align the central dot on an imaginary straight line between the paracentral points by tapping the arrow keys (Figure 1). This assignment was repeated 12 times in 4 different axes until the test was completed and the patient received a score between zero and 100. A higher score should be indicative of less metamorphopsia in contrast to the M-chart where a higher score should indicate more pronounced metamorphopsia. The SHT covers 4 axes with fixation dots at different positions while the M-chart covers 2 axes with a central single fixation dot (Figure 1).

### SD-OCT Biomarker Evaluations

**Figure 2.**
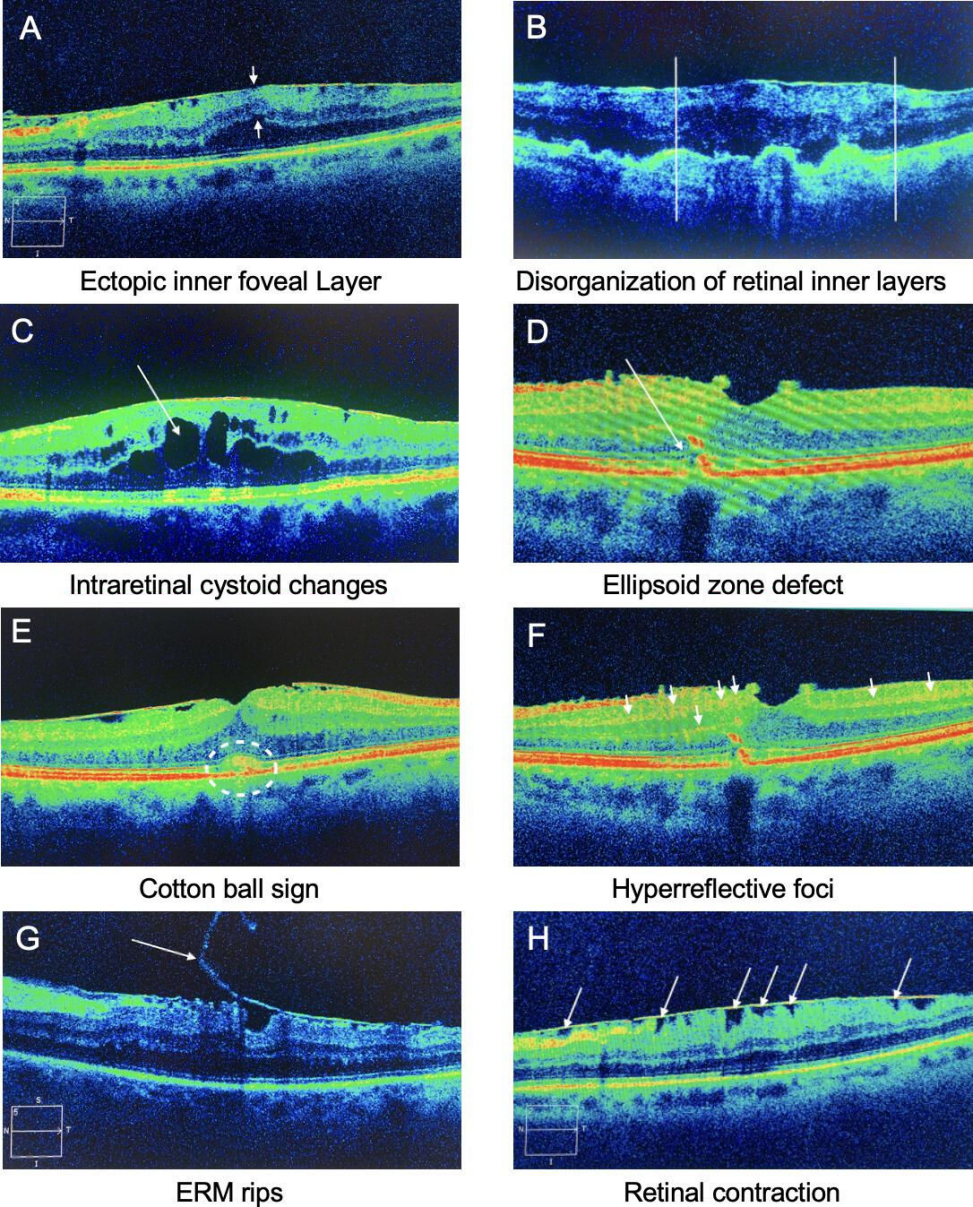
Spectral-domain optical coherence tomography (SD-OCT) biomarkers A–H (pictures taken and modified by Amon D). (A) Ectopic inner foveal layer is the presence of a continuous hypo- or hyperreflective lamina expanding from the inner nuclear layer and inner plexiform layer over the foveal zone as indicated by the arrows. (B) Disorganisation of retinal inner layers is present when the borders between the inner retinal layers are not recognisable as indictated between the two lines. (C) Intraretinal cystoid changes are fluid accumulations within the retina that can be recognised as hyporeflective spaces on OCT scans as indicated by the arrow. (D) The ellipsoid zone (EZ) is a hyperreflective region built between the interface of inner and outer photoreceptor segments and disruption can be seen as discontinuation of the hyperreflective EZ band as indicated by the arrow. (E) Cotton ball sign is defined as a round or diffuse hyperreflective area between the EZ and the interdigitation zone within the centre of the fovea as indicated by the circle. (F) Hyperreflective foci appear as small, highly reflective dots scattered within different layers of the retina as indicated by the arrows. (G) Epiretinal membrane rips are depicted as a torn edge of the ERM with a scrolled flap as indicated by the arrow. (H) Retinal contraction appears as wrinkling of the underlying retina caused by contraction of the ERM as indictaed by the arrows. ERM: epiretinal membrane.

As a secondary outcome this study evaluated the correlation of metamorphopsia scores and OCT biomarkers. OCT biomarkers are specific changes in retinal morphology with possible influence on postsurgical outcome. The preoperative SD-OCT images were evaluated by 2 independent graders (AD and SA) who were ophthalmology residents trained for OCT diagnosis. The readings of the 2 readers were compared and in case of discrepancies, the third reader, an experienced retina specialist made a final decision. The images were screened regarding the presence or absence of the following SD-OCT biomarkers: ectopic inner foveal layer, disorganization of retinal inner layers, intraretinal cystoid changes, ellipsoid zone defect, cotton ball sign, hyperreflective (HR) foci, ERM rips, and retinal contraction (Figure 2). Central macular thickness was also included in the analysis.

### Subjective Perception of Metamorphopsia

This study additionally tried to quantify the subjective perception of metamorphopsia using a telephone questionnaire. We postoperatively asked patients to grade their pre- and postoperative perception of metamorphopsia on a scale ranging from “0” indicating no distortion to “5” indicating severe distortion. Correlations between the subjective grades and standardized metamorphopsia scores were evaluated.

### Statistical Analysis

Statistical analyses were conducted using SPSS (version 28; IBM SPSS Statistics). Level of significance was defined as a *P* value below .05. The Shapiro-Wilk test was used to test for normality. Reproducibility for the SHT and M-chart test was evaluated using intraclass correlation coefficients (ICCs) between 3 consecutive measurements pre- and postoperatively. We used the interpretation by Koo and Li [8] suggesting that ICC values below 0.5 are indicative of poor reliability, values between 0.5 and 0.75 are indicative of moderate reliability, values between 0.75 and 0.9 are indicative of good reliability, and values greater than 0.90 are indicative of excellent reliability. Correlations between the mean (mean of 3 examinations) SHT scores, mean MH, mean MV, the sum of mean MH and mean MV (MH+MV mean), and DCVA were calculated with a bivariate correlation. Paired 2-tailed *t* tests were applied to analyze the potential difference between preoperative and postoperative SHT and M-chart scores as well as DCVA. The *t* tests and bivariate correlation were used for the evaluation of the relationship between metamorphopsia scores and postoperative positive Amsler test, OCT biomarkers, and subjective metamorphopsia scores. Correlation coefficients were calculated using the Pearson correlation (*r*). Classification of the magnitude of correlation by Wuensch and Evans [9] was used interpreting an *r* value below 0.20 as very weak, *r* values between 0.20 and 0.39 as week, *r* values between 0.40 and 0.59 as moderate, *r* values between 0.60 and 0.79 as strong, and *r* value above 0.80 as very strong correlation.

## Results

### Demographic Data and Visual Acuity

A total of 30 patients were included into this study. Three of the 30 patients who had undergone preoperative examinations and surgery for iERM were lost to postoperative follow-up as they did not wish to participate in the postoperative study examinations and were therefore excluded from the final analysis. The mean age of our patient cohort was 71.2 (SD 8.2) years (Table 1). Regarding lens status, 12 patients were pseudophakic preoperatively and 15 were phakic preoperatively. Of the 15 preoperative patients with phakia, 12 patients underwent combined phacovitrectomy. Surgery resulted in a mean DCVA change of 15.3 (SD 9.3) ETDRS letters, with a minimum of −7 letters and a maximum change of 35 letters (*P*<.001; Table 2). The mean preoperative DCVA was 62.3 (SD 11.6) ETDRS letters compared with the mean postoperative DCVA with 77.6 (SD 8.2) letters. No significant correlations between the metamorphopsia scores and change in DCVA were found (Table 3).

**Table 1.** Demographic data (N=27).

Characteristics	Values
Age (years)	
Mean (SD)	71.2 (8.2)
Maximum	88
Minimum	54
Sex, n (%)	
Female	9 (33)
Male	18 (66)
Eye, n (%)	
Right eye	12 (44)
Left eye	15 (55)
Lens status, n (%)	
Preoperative phakia	15 (56)
Preoperative pseudophakia	12 (44)
Combined phacovitrectomy	12 (44)

**Table 2.** Visual acuity outcomes (Early Treatment Diabetic Retinopathy Study letters).

	Preoperative DCVA^a^	Postoperative DCVA	Change in DCVA
Mean (SD)	62.3 (11.6)	77.6 (8.2)	15.3 (9.3)
Maximum	82	90	35
Minimum	40	60	–7

a DCVA: distance-corrected visual acuity.

**Table 3.** Correlations between metamorphopsia scores and change in distance-corrected visual acuity.

	Pearson *r*	*P* value
SHT^a^ mean preoperative	–0.17	.40
MH^b^ mean preoperative	–0.16	.41
MV^c^ mean preoperative	0.03	.87
MH+MV mean preoperative	–0.11	.59
SHT mean postoperative	–0.26	.18
MH mean postoperative	0.14	.49
MV mean postoperative	0.25	.22
MH+MV mean postoperative	0.20	.32

a SHT: smartphone-based hyperacuity test.

b MH: horizontal M-chart.

c MV: vertical M-chart.

### Metamorphopsia Test Results

#### Change of Metamorphopsia After Surgery

**Figure 3.**
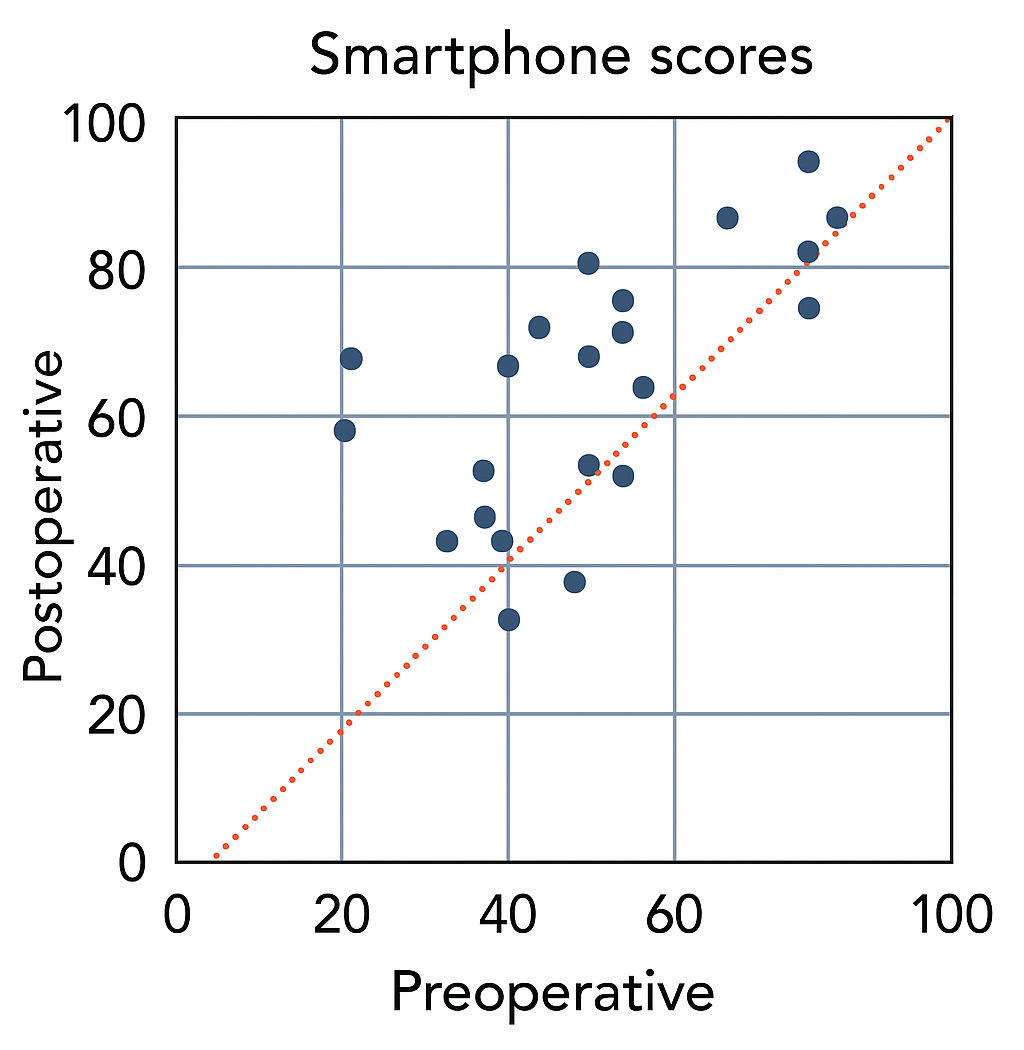
Scatter plot depicting dots above the 45° line indicate patients who had improved smartphone-based hyperacuity test scores after epiretinal membrane surgery.

Patients had significantly higher (*r*=0.69; *P*<.001) postoperative SHT scores compared with scores before surgery and a strong correlation between the 2 variables was found (Figure 3). The preoperative mean SHT score was 55.2 (SD 18.9) compared with 63.5 (SD 16.3) postoperatively resulting in a difference of 8.30 points. The improvement of M-chart scores, however, did not prove to be significant and the preoperative and postoperative values showed a weak correlation. The mean preoperative MH changed from 0.58 (SD 0.39) to 0.43 (SD 0.42) (*r*=0.37; *P*=.06) with a difference of 0.15, mean preoperative MV from 0.50 (SD 0.24) to 0.43 (SD 0.36) (*r*=0.18; *P*=.36) with a difference of 0.07, and mean preoperative MH+MV from 1.08 (SD 0.52) to 0.87 (SD 0.73) (*r*=0.25; *P=*.20) with a difference of 0.21 (Table 4).

**Table 4.** Correlations between metamorphopsia scores.

Pairs of metamorphopsia scores	Mean (SD)	Pearson *r*	*P* value
Pair 1			
SHT^a^ mean preoperative	55.22 (18.85)	0.69	<.001
SHT mean postoperative	63.52 (16.26)		
Pair 2			
MH^b^ mean preoperative	0.58 (0.39)	0.37	.06
MH mean postoperative	0.43 (0.42)		
Pair 3			
MV^c^ mean preoperative	0.50 (0.24)	0.18	.83
MV mean postoperative	0.43 (0.36)		
Pair 4			
MH+MV mean preoperative	1.08 (0.52)	0.25	.21
MH+MV mean postoperative	0.87 (0.73)		
Pair 5			
SHT mean preoperative	55.22 (18.85)	−0.18	.369
MH mean preoperative	0.58 (0.39)		
Pair 6			
SHT mean preoperative	55.22 (18.85)	0.01	.97
MV mean preoperative	0.50 (0.24)		
Pair 7			
SHT mean preoperative	55.22 (18.85)	−0.13	.51
MH+MV mean preoperative	1.08 (0.52)		
Pair 8			
MH mean preoperative	0.58 (0.39)	0.32	.10
MV mean preoperative	0.50 (0.24)		
Pair 9			
SHT mean postoperative	63.52 (16.26)	−0.14	.48
MH mean postoperative	0.43 (0.42)		
Pair 10			
SHT mean postoperative	63.52 (16.26)	−0.20	.32
MV mean postoperative	0.43 (0.36)		
Pair 11			
SHT mean postoperative	63.52 (16.26)	−0.18	.37
MH+MV mean postoperative	0.87 (0.73)		
Pair 12			
MH mean postoperative	0.43 (0.42)	0.74	<.001
MV mean postoperative	0.43 (0.36)		

a SHT: smartphone-based hyperacuity test.

b MH: horizontal M-chart.

c MV: vertical M-chart.

#### Reproducibility of Metamorphopsia Tests

The ICCs of all the metamorphopsia measurements showed good reliability (Table 5). The horizontal and vertical M-chart tests achieved higher scores than the SHT showing excellent reliability before and after surgery.

**Table 5.** Intraclass correlation coefficients of metamorphopsia tests.

Metamorphopsia test	Intraclass correlation coefficient
SHT^a^ preoperative	0.87
MH^b^ preoperative	0.96
MV^c^ preoperative	0.94
SHT postoperative	0.82
MH postoperative	0.97
MV postoperative	0.93

a SHT: smartphone-based hyperacuity test.

b MH: horizontal M-chart.

c MV: vertical M-chart.

#### Correlations Between SHT and M-Chart

Correlations between the SHT and the M-chart were found to be very weak and not significant preoperatively as well as postoperatively. The correlation coefficients of the metamorphopsia scores and the corresponding levels of significance are listed in Table 4.

#### Correlation Between Metamorphopsia Scores and Amsler Grid

In 7 patients, metamorphopsia was not detected on the Amsler grid after surgery compared with 20 patients who still showed a positive postoperative Amsler test. The group of patients with a positive Amsler test after surgery showed significantly (*r*=−0.46; *P*=.02) worse postoperative SHT scores than the group without (Table 6). The mean SHT score of the patients with metamorphopsia detected on the Amsler grid after surgery was 59.2 (SD 15.3) compared with 75.7 (SD 12.9) of the patients with a negative Amsler test resulting in a difference of 16.7 between these groups. The group of patients with a postoperative positive Amsler test also showed significantly worse MV (*r*=0.39; *P*=.04) and MH+MV (*r*=0.39; *P*=.047) scores while the MH score was not significantly different between the groups (*r*=0.33; *P*=.09).

**Table 6.** Correlations between metamorphopsia scores and Amsler grid.

Postoperative	Amsler +	N	Mean (SD)	Pearson *r*	*P* value
SHT^a^ score	Negative	7	75.9 (12.9)	−0.46	.02
	Positive	20	59.2 (15.3)		
MH^b^ score	Negative	7	0.2 (0.3)	0.33	.09
	Positive	20	0.5 (0.4)		
MV^c^ score	Negative	7	0.2 (0.3)	0.39	.04
	Positive	20	0.5 (0.4)		
MH+MV score	Negative	7	0.4 (0.5)	0.39	.047
	Positive	20	1.0 (0.7)		

a SHT: smartphone-based hyperacuity test.

b MH: horizontal M-chart.

c MV: vertical M-chart.

#### Correlation Between Metamorphopsia Scores and SD-OCT Biomarkers

Central macular thickness (*r*=−0.44; *P*=.02; *r*=−0.46; *P*=.02) and intraretinal cysts (*r*=−0.72; *P*<.001; *r*=−0.65; *P*<.001) proved to be significantly associated with pre- and postoperative SHT scores and HR foci showed a significant correlation with the postoperative SHT score (*r*=0.44; *P*=.02). Regarding the M-chart scores, the disorganization of retinal inner layers (*r*=0.68, *P*<.001; *r*=0.58, *P*=.002) and ellipsoid zone defect (*r*=0.49, *P*=.01; *r*=0.48, *P*=.01) showed significant associations with the preoperative vertical M-chart score (*r*=0.68; *P*<.001) and the preoperative MH+MV score (*r*=0.58; *P*=.002). The other biomarkers were not significantly associated with any of the metamorphopsia scores and correlations proved to be very weak to weak. Detailed results of the biomarker readings can be found in the Multimedia Appendix 1.

#### Correlation Between Metamorphopsia Scores and Subjective Metamorphopsia Perception

Out of 21 patients surveyed, 19 reported a subjective improvement in metamorphopsia after surgery. The mean subjective scores improved by 2.2 points from 3.7 (SD 0.9) preoperatively to 1.5 (SD 1.3) postoperatively, showing a significant correlation (*r*=0.55; *P*=.01;). The preoperative (*r*=0.58; *P*=.01) and postoperative (*r*=0.81; *P*<.001) subjective metamorphopsia scores correlated significantly with postoperative M-Chart scores, but not significantly with SHT scores. Patients with postoperative positive Amsler tests reported significantly more severe subjective metamorphopsia scores (*r*=0.49; *P*=.02).

## Discussion

### Principal Findings

The aim of this study was to estimate the correlation between the SHT (Alleye app) and a conventional printed chart (M-chart) for metamorphopsia quantification before and after vitrectomy with membrane peeling for patients with iERM.

The SHT has mainly been studied for other retinal diseases and this study is the first to ever investigate the SHT in patients with iERM. It is the first mobile app to provide a quantitative metamorphopsia result. Contrary to the vertical and horizontal scores of the M-chart, the SHT provides a single quantitative score including 4 axes instead of 2.

The SHT scores significantly (*r*=0.69; *P*<.001) improved from 55.2 (SD 18.9) preoperatively to 63.5 (SD 16.3) after surgery resulting in an improvement of 8.3 points. The mean postoperative M-chart scores also improved after surgery, but were not statistically significant. The significant improvement of postoperative SHT scores suggests that patients with metamorphopsia due to iERM may benefit from surgery and the SHT can potentially be a software to quantify these results. Clinicians routinely do not test for metamorphopsia, despite its relevance for surgical decisions. Kinoshita et al [3,10] found a significant correlation between preoperative and postoperative M-chart scores proposing the consideration of surgery before severe worsening of metamorphopsia and a stage where it cannot be completely resolved. Testing macular function with visual acuity alone is often insufficient, as it fails to capture metamorphopsia, a subjective perception that is challenging to measure objectively. In our study, both the SHT and the M-chart demonstrated good reliability, as shown by their ICCs. However, the SHT may offer a more comprehensive assessment by covering 4 retinal axes, whereas the M-chart evaluates only 2. It is important to note that this study did not aim to test the superiority of the SHT over the M-chart but was a first attempt to evaluate the SHT in patients with iERM aiming to identify a practical tool that enables efficient and reliable measurement of metamorphopsia in clinical practice. The tests provide complementary but distinct insights into the severity of metamorphopsia, and their scores should not be considered directly comparable. The SHT is primarily used for detecting macular edema. This was the first attempt to evaluate the SHT in patients with ERM, providing initial insights into its potential use for this condition. In addition, the SHT, being a more practical and digital tool, could offer significant advantages in clinical practice. The SHT’s ability to provide a unified score, enable remote monitoring, and incorporate gamification may improve patient engagement and reduce unnecessary clinic visits.

Of interest, the results of our analysis showed a very weak and not significant association between the SHT and the M-chart. Since the M-chart includes only 2 axes, horizontal and vertical, while the SHT includes the horizontal, vertical, and oblique axes, there is a discrepancy between the examined retinal axes. A key limitation of this study is the inability to analyze individual horizontal, vertical, and oblique axes measured by the SHT, which could allow for a more direct comparison with the M-chart, which evaluates horizontal and vertical axes separately. To minimize the discrepancy between the examined axes, we combined the 2 M-chart axes and calculated the sum of the mean vertical M-chart score and mean horizontal M-chart score. The 2 axes included in 1 score, however, also did not significantly correlate with the SHT score. Analyses of the SHT’s individual axes might help clarify whether the weak correlation observed between the 2 tests is due to differences in the retinal areas examined. However, this was not feasible due to the proprietary design of the SHT, which provides only a combined score rather than axis-specific data. This limitation has been communicated to the developers, and we recommend incorporating functionality for individual axis analysis in future updates to enhance the SHT’s scientific use. Despite this constraint, studies have demonstrated the reliability and practicality of the SHT. Studies reported high ICCs, diagnostic reliability for monitoring macular function, good patient adherence and usability for remote monitoring, and a low threshold for use, making it accessible and effective in real-world settings [4,11-13]. These advantages position the SHT as a practical alternative for assessing metamorphopsia in clinical practice, even without detailed axis-specific data. In addition, the SHT’s combined score approach may simplify the interpretation of metamorphopsia as a general symptom of macular dysfunction, supporting its use in patient-centered care. Nevertheless, the mapping of distorted vision to a specific axis may additionally be of use since the horizontal M-chart score tends to improve to a larger extent than the vertical score that arises in later stages suggesting that once the vertical distortion is present, it is less likely to resolve compared with horizontal metamorphopsia [10]. Our study as well as the study by Kinoshita et al [3,10] showed that the baseline MH score was higher than the MV score. The vertical plasticity may be greater than that for the horizontal retina since the axons of the retinal ganglion cells run horizontally rather than vertically in the posterior pole in addition to the optic disc that might also reduce horizontal displacement in the posterior pole. Since vertical contraction is perceived as horizontal metamorphopsia and vice versa, the horizontal M-chart scores might be higher than the vertical scores [14,15]. The prognostic properties of individual metamorphopsia axes may be of importance in advising patients and giving them a realistic prognosis for postoperative outcomes. Schmid et al [4] claimed that when using the SHT, patients actively need to align a central point on an imaginary straight line and the outer points remain stable in the paracentral visual field thereby ensuring that patients fixate on the moving dot and avoiding saccades to the outer areas. This should ensure a proper fixation for metamorphopsia testing. In passive tests such as the M-chart or the Amsler grid, where patients are shown different lines or grids and asked about their perception of them, fixation on paracentral areas is more likely, which may lead to the conclusion of lower reproducibility. In our study, however, all metamorphopsia tests showed a good reliability (Table 5) and the M-chart even achieved higher scores than the SHT proving excellent reliability before and after surgery. This underlines the findings by Matsumoto et al [16] reporting good reliability of M-charts and intraindividual variation to be within 1 line (±0.1 score).

Regarding associations of metamorphopsia scores with the Amsler grid, almost a third of the patients had improved metamorphopsia after surgery when testing with the Amsler grid. The group of patients with a positive Amsler test after surgery showed significantly worse postoperative SHT scores (*r*=−0.46; *P*=.02), MV scores (*r*=0.40; *P*=.04), and MH+MV scores (*r*=0.39; *P*=.047) than the group without. The results suggest that a persistent positive Amsler test may be a reliable predictor of poor SHT scores and therefore outcome.

Postoperative mean visual acuity improved by 3 lines and no significant correlation between DCVA and metamorphopsia was found which is in accordance with literature [3,17-19]. Lens status revealed that 12 patients were pseudophakic preoperatively, while 15 were phakic, of whom 12 underwent combined phacovitrectomy. Since visual acuity was not the main focus of this study, the lens status was not further emphasized in the analysis. Metamorphopsia seems to be independent of visual acuity and an important symptom contributing to quality of vision.

Regarding the OCT biomarkers, a larger central macular thickness, the presence of intraretinal cysts, and HR foci were significantly associated with postoperative SHT scores. These findings suggest that these biomarkers may have prognostic properties for metamorphopsia outcomes after ERM surgery. This is particularly relevant in clinical decision-making, as identifying OCT biomarkers associated with postoperative visual distortion could help guide surgical timing and set realistic patient expectations. To validate our findings, larger studies and longer follow-up periods are necessary. These studies should further investigate the relationship between objective metamorphopsia scores, OCT biomarkers, and subjective metamorphopsia perception. Such research could refine the prognostic use of OCT findings and contribute to the development of comprehensive assessment tools that integrate objective measures with subjective experiences. Ultimately, this could lead to more tailored surgical interventions and improved patient outcomes in ERM management.

During the postoperative follow-up visits of our study, we frequently examined content patients stating that their visual distortion had highly improved and asking whether their scores had improved in an objective manner. This observation underlined the importance of an individual’s perception of vision. Our study demonstrated that subjective perception of metamorphopsia in patients with iERM can improve with surgery and patients with a higher preoperative degree of metamorphopsia also suffer from more severe metamorphopsia postoperatively. Detailed results on the subjective outcomes are given in Multimedia Appendices 2 and 3.

An important aspect to consider with the SHT is the possibility for patients to quantitatively monitor the progression of metamorphopsia at home in a comfortable setting and compare their results with their last examinations. Interactive tests and the gamification of home-monitoring tasks can lead to a higher patient motivation to take ownership of their eye health as well as better protection of sight [20,21]. Smartphone apps can also serve as a tool for patients to remotely view their health record information [22]. The newest version of the SHT is designed to serve as a digital companion for patients to have a better overview of their retinal disease and keep them motivated to follow the treatment program. The novel features of the SHT enable patients and clinicians to collect data, manage appointments, and document diagnoses, medical results, visual acuity, injections, and subjective visual impairment [23]. Since many people already own a smartphone, the implementation of the SHT is rather straightforward. In our study, we included a large age range between 54 and 88 years and none of the patients needed extra help to perform the SHT after a short introduction at the beginning of the test. It is important, however, that patients who do not qualify for home monitoring are not disadvantaged and do not receive insufficient care for their eye health. Research suggests that smartphone-based vision monitoring is accessible across diverse population groups with varying levels of digital proficiency and social deprivation, making it a viable and reliable tool for monitoring clinical progression [24].

Currently, no established thresholds exist to define clinically significant changes in SHT scores or to determine when patients should seek medical advice. Further studies are necessary to address these gaps and to establish evidence-based benchmarks for the SHT’s use in clinical decision-making. Previous research has provided M-chart–based thresholds for surgical indications; however, these cutoff values have not been adopted in clinical practice. Kinoshita et al [3] suggested that preoperative MH scores between 0.5 and 1.7 or MV scores between 0.5 and 0.9 could indicate the need for surgery. While strict cutoff values may be challenging in individual cases, quantifying metamorphopsia can enhance the decision-making processes. The SHT can complement traditional tools by providing additional, objective data to support surgical planning, facilitate triaging, and monitor disease progression.

Compared with the conventional printed M-chart for metamorphopsia quantification, the SHT offers several advantages that align with modern, patient-centered health care approaches. The SHT provides a unified score that supports longitudinal monitoring and examines more retinal axes, offering a more comprehensive assessment of metamorphopsia. In addition, the incorporation of gamification enhances patient engagement and adherence, transforming a clinical task into an interactive and motivating experience for patients. These features may make the SHT a practical tool for continuous care, particularly for patients with ERM who require long-term monitoring with frequent checkups. Patients with ERM undergo follow-ups spanning several years, with frequent visits to monitor disease progression or assess the need for surgical intervention. A reliable and easily accessible tool such as the SHT could serve as a valuable monitoring score to detect changes or dynamics in metamorphopsia over time. Its capability for remote testing can further reduce the need for in-person visits, supporting telemedicine pathways and addressing the increasing demand for accessible health care solutions. Moreover, the SHT can be particularly beneficial for patients in underserved areas, those with mobility limitations, or in regions with limited physician availability, ensuring broader access to effective disease monitoring. While the SHT has demonstrated its use in other retinal conditions, its relevance in the context of ERM has yet to be fully evaluated. These attributes suggest that the SHT could play a key role in the evolution of digital health tools for monitoring metamorphopsia and guiding complex surgical decision-making in patients with ERM.

This study represents a foundational step in evaluating the SHT’s app in ERM, emphasizing the need for further research to validate its clinical relevance and establish its role in patient-centered ophthalmologic care.

### Limitations

Limitations of this study include the small sample size and the limited follow-up time as metamorphopsia tends to improve for a longer period of about 12 months after ERM surgery and a small number of patients is a limitation for the validity of the test [3,10]. To date, it has unfortunately not been possible to retrieve the results of the individual axes of the SHT separately as it would be of great interest to depict what the diagonal planes measure and calculate their correlations with the horizontal and vertical planes of the M-Chart. Without this breakdown of SHT results, the hypothesis that the discrepancy in examined retinal axes may be a reason why the scores of the 2 tests did not significantly correlate with each other cannot be fully proven. Due to the exploratory nature of this study, we could not perform a formal sample size calculation. Another confounder of this study may be patient motivation and character as well as a learning effect with repeated testing. The potential influence of learning effects on repeated testing with the SHT and M-chart is an important consideration for interpreting the study findings. While we assessed test-retest reliability for both tools, we did not explicitly analyze learning effects. Previous research on the SHT, including a study by Faes et al [12], demonstrated its excellent usability with a median system usability score of 90. This indicates that most users found the app intuitive and easy to learn, suggesting that the influence of learning on SHT results may be minimal. Furthermore, the digital nature of the SHT, combined with its interactive and standardized testing process, reduces the likelihood of variability due to user fatigue or concentration, issues that are more likely to affect manual tools such as the M-chart. Incorporating design elements that prioritize user-friendly interfaces, as seen with the SHT, can help maintain adherence and reduce the impact of learning effects in mobile health tools. Future studies could further investigate the influence of learning on SHT performance. As not all patients were pseudophakic after surgery, the improvement of DCVA may have been influenced by concomitant cataract surgery; however, visual acuity was not a main outcome of this study. Our study was conducted at a single center and all surgeries were performed by the same retina surgeon thereby increasing reproducibility.

### Conclusions

The study was the first to ever investigate the SHT in patients with iERM. We showed that quantitative data provided by the SHT significantly improved after membrane peeling suggesting that patients with metamorphopsia due to iERM can benefit from surgery, and this application may potentially be a software to quantify metamorphopsia in patients with iERM. The metamorphopsia scores of the SHT showed a very weak and insignificant association with the M-chart scores. It would be of great interest to depict what the diagonal planes of the SHT measure and calculate their correlations with the horizontal and vertical planes of the M-chart to validate whether the poor correlation between the 2 tests may be explained by their discrepancy in examined retinal areas. The SHT may serve as a practical, accessible, and patient-centered tool for monitoring metamorphopsia, supporting long-term care and decision-making for patients with ERM, particularly in telemedicine and underserved settings. While this study lays the groundwork for future research, further studies including the breakdown of metamorphopsia axes of the SHT as well as a larger sample size and longer follow-up period are required to validate our results.

## Supplementary material

10.2196/60959 Multimedia Appendix 1 Spectral-domain optical coherence tomography biomarker readings of the pilot study.

10.2196/60959 Multimedia Appendix 2 Correlations between preoperative spectral-domain optical coherence tomography biomarkers and preoperative metamorphopsia scores.

10.2196/60959 Multimedia Appendix 3 Correlations between preoperative spectral-domain optical coherence tomography biomarkers and postoperative metamorphopsia scores.

## Abbreviations

DCVA: distance-corrected visual acuity
ERM: epiretinal membrane
HR: hyperreflective
ICC: intraclass correlation coefficient
iERM: idiopathic epiretinal membrane
ILM: internal limiting membrane
MH: horizontal M-chart
MV: vertical M-chart
OCT: optical coherence tomography
SD-OCT: spectral-domain optical coherence tomography
SHT: smartphone-based hyperacuity test
