# Supplementary material for: Quantification of Metamorphopsia Using a Smartphone-Based Hyperacuity Test in Patients With Idiopathic Epiretinal Membranes: Prospective Observational Study
Source: JMIR Perioper Med. 2025 Apr 17;8:e60959. doi: 10.2196/60959 (PMC12021372; doi:10.2196/60959)
Supplement: Multimedia Appendix 1 [file periop-v8-e60959-s001.docx]

Multimedia Appendix 1 SD-OCT biomarker readings of the pilot study.

| SD-OCT Biomarker | Patients (n) | Frequency (absolute), n | Percent, % | Interobserver Reliability |
| --- | --- | --- | --- | --- |
| EIFL | 26 | 21 | 80 | 0.31 |
| DRIL | 27 | 3 | 11 | 0.82 |
| ICC | 27 | 13 | 48 | 0.89 |
| EZ defect | 27 | 1 | 3 | 1 discrepancy |
| Cotton ball sign | 27 | 7 | 25 | 0.72 |
| HR foci | 27 | 13 | 48 | 0.54 |
| ERM rips | 27 | 8 | 29 | 0.90 |
| Retinal contraction | 27 | 26 | 96 | 4 discrepancies |
| CMT | 27 | Mean ± SD | Minimum | Maximum |
|  |  | 500.74 ± 73.10 | 360 µm | 655 µm |
