# Supplementary material for: Quantification of Metamorphopsia Using a Smartphone-Based Hyperacuity Test in Patients With Idiopathic Epiretinal Membranes: Prospective Observational Study
Source: JMIR Perioper Med. 2025 Apr 17;8:e60959. doi: 10.2196/60959 (PMC12021372; doi:10.2196/60959)
Supplement: Multimedia Appendix 2 [file periop-v8-e60959-s002.docx]

Multimedia Appendix 2 Correlations between preoperative SD-OCT biomarkers and preoperative metamorphopsia scores.

| Biomarker  preop | SHT mean preop | | MH mean preop | | MV mean preop | | MH+MV mean preop | |
| --- | --- | --- | --- | --- | --- | --- | --- | --- |
|  | r | *P*-value | r | *P*-value | r | *P*-value | r | *P*-value |
| EIFL | -.05 | .83 | .14 | .50 | .36 | .69 | .27 | .18 |
| DRIL | -.38 | .05 | .35 | .07 | .68 | <.001* | .58 | .002* |
| ICC | -.72 | <.001* | .13 | .53 | .13 | .53 | .15 | .44 |
| EZ defect | -.17 | .41 | .34 | .09 | .49 | .01* | .48 | .01* |
| Cotton ball sign | .03 | .88 | .14 | .49 | .26 | .20 | .22 | .27 |
| HR foci | -.31 | .11 | .34 | .08 | -.09 | .64 | .21 | .29 |
| ERM rips | -.10 | .61 | .32 | .10 | .21 | .29 | .34 | .09 |
| Retinal contraction | -.03 | .87 | .02 | .91 | .09 | .67 | .06 | .78 |
| CMT | -.44 | .02* | .17 | .40 | .46 | .02* | .34 | .08 |
