# Supplementary material for: Quantification of Metamorphopsia Using a Smartphone-Based Hyperacuity Test in Patients With Idiopathic Epiretinal Membranes: Prospective Observational Study
Source: JMIR Perioper Med. 2025 Apr 17;8:e60959. doi: 10.2196/60959 (PMC12021372; doi:10.2196/60959)
Supplement: Multimedia Appendix 3 [file periop-v8-e60959-s003.docx]

Multimedia Appendix 3 Correlations between preoperative SD-OCT biomarkers and postoperative metamorphopsia scores.

| Biomarker  preop | SHT mean postop | | MH mean postop | | MV mean postop | | MH+MV mean postop | |
| --- | --- | --- | --- | --- | --- | --- | --- | --- |
|  | r | *P*-value | r | *P*-value | r | *P*-value | r | *P*-value |
| EIFL | -.07 | .75 | .07 | .73 | .15 | .467 | .13 | .58 |
| DRIL | -.19 | .35 | .29 | .14 | .19 | .341 | .27 | .18 |
| ICC | -.65 | <.001* | -.05 | .80 | -.12 | .551 | -.09 | .66 |
| EZ defect | -.25 | .20 | .33 | .09 | .00 | 1.00 | .19 | .34 |
| Cotton ball sign | .12 | .57 | .16 | .43 | .08 | .69 | .13 | .51 |
| HR foci | .44 | .02* | .31 | .12 | .22 | .27 | .29 | .15 |
| ERM rips | -.00 | .98 | .30 | .14 | .21 | .30 | .27 | .17 |
| Retinal contraction | -.12 | .55 | -.00 | 1.00 | .06 | .78 | .04 | .89 |
| CMT | -.46 | .02* | .31 | .11 | .27 | .18 | .31 | .11 |

# 
